# Actin-Like Protein 6A as an Oncogene and Therapeutic Target in Cancer

**DOI:** 10.7150/ijms.113736

**Published:** 2025-06-12

**Authors:** Guo-Bin Song, Lin Xiang, Tian Peng, Ya-Nan Li, Hou-Qun Ying, Xue-Xin Cheng

**Affiliations:** Jiangxi Province Key Laboratory of Immunology and Inflammation, Jiangxi Provincial Clinical Research Center for Laboratory Medicine, Department of Clinical Laboratory, The Second Affiliated Hospital, Jiangxi Medical College, Nanchang University, Nanchang 330006, Jiangxi, People's Republic of China.

**Keywords:** ACTL6A, cancer, chromatin remodeling, tumor progression, therapeutic target

## Abstract

ACTL6A, a core subunit of the SWI/SNF chromatin remodeling complex, has emerged as a critical oncogenic driver across multiple malignancies. Recent studies reveal that aberrant ACTL6A overexpression promotes tumor initiation, progression, and metastasis by orchestrating chromatin remodeling, transcriptional reprogramming, and crosstalk with key signaling pathways (e.g., Hippo/YAP, Notch, and PI3K/AKT). This review systematically synthesizes evidence from *in vitro*, *in vivo*, and clinical studies spanning hepatocellular carcinoma, breast cancer, glioblastoma, and 10 other cancer types, highlighting ACTL6A's dual role as a chromatin remodeler and an independent oncogenic effector. Key mechanisms include sustaining cancer stemness, suppressing apoptosis, enhancing DNA repair, and driving metabolic reprogramming. Clinically, ACTL6A overexpression correlates with advanced tumor stage, therapy resistance, and poor prognosis, positioning it as a promising prognostic biomarker and therapeutic target. We further discuss emerging strategies to inhibit ACTL6A (e.g., siRNA, small-molecule inhibitors) and propose combinatorial approaches to overcome drug resistance. By integrating multi-omics data and preclinical models, this review not only clarifies ACTL6A's context-dependent oncogenic networks but also bridges mechanistic insights to translational challenges, offering a roadmap for future research and therapeutic development.

## Introduction

Cancer progression is driven by the dysregulation of molecular networks controlling proliferation, survival, and differentiation.^1^ While canonical oncogenes (e.g., *MYC*, *RAS*) and tumor suppressors (e.g., *TP53*) have been extensively studied, recent advances highlight chromatin remodeling complexes as central players in tumorigenesis.^2^-^4^ Among these, the SWI/SNF complex, an ATP-dependent chromatin remodeler, is frequently mutated or dysregulated in cancers.^5^ Actin-like protein 6A (ACTL6A), a core subunit of SWI/SNF and other chromatin-modifying complexes (e.g., INO80, NuA4/TIP60), has emerged as a unique oncogenic driver with dual roles: maintaining chromatin architecture and independently rewiring transcriptional programs to fuel malignancy.^6^,^7^

Unlike classical oncoproteins, ACTL6A exerts context-dependent effects across cancers.^8^,^9^ It sustains stemness in hepatocellular carcinoma (HCC), drives metabolic reprogramming in ovarian cancer, and confers therapy resistance in advanced prostate cancer.^10^-^12^ Mechanistically, ACTL6A coordinates with transcription factors (e.g., YAP, MYC) to amplify oncogenic signaling while suppressing differentiation and apoptosis.^13^-^15^ Despite its emerging significance, critical gaps remain:

**Mechanistic heterogeneity**: How does ACTL6A achieve divergent functions (e.g., pro-survival vs. pro-metastatic) in different cancer types?

**Therapeutic vulnerability**: Can ACTL6A-targeted strategies overcome resistance to conventional therapies?

**Clinical translation**: What biomarkers predict ACTL6A dependency, and how to mitigate off-target effects?

Current reviews on ACTL6A focus narrowly on its role in chromatin remodeling, lacking integration of multi-omics data and cross-cancer analyses. This review addresses these gaps by synthesizing evidence from 15 cancer types, including solid tumors (e.g., breast, lung) and rare malignancies (e.g., rhabdomyosarcoma). We dissect ACTL6A's mechanisms through three lenses: Biological Functions: Encompassing its influence on cell proliferation, differentiation, and apoptosis, as well as its participation in key signaling cascades; Normal Physiological Processes: Looking into its function in embryonic development, tissue homeostasis, and immune regulation;Oncogenic Role Across Cancers: Analyzing how it promotes tumor initiation, progression, and metastasis in diverse cancer settings, along with its correlation with patient prognosis.

By bridging mechanistic insights with translational challenges, this work aims to redefine ACTL6A as a linchpin of cancer adaptability and provide a roadmap for precision oncology strategies.

## Biological Functions of ACTL6A

ACTL6A is a key member of the actin protein family and serves as an essential auxiliary subunit in several chromatin remodeling complexes, including the SWI/SNF-like BAF complex,^5^ the INO80 complex,^16^ and the NuA4/TIP60 acetyltransferase complex.^17^ Firstly, the SWI/SNF-like BAF (Brg1/Brm-associated factors) complex is an ATP-dependent chromatin remodeling complex that utilizes the energy from ATP hydrolysis to modify chromatin structure and regulate gene expression.^18^ ACTL6A interacts with the SWI/SNF complex to activate the Brg1 ATPase protein. Brg1, a chromatin remodeling factor in the SWI/SNF family, employs ATP hydrolysis to remodel chromatin, which allows specific DNA sequences to become more accessible to transcription factors and other regulatory proteins.^19^ This remodeling is crucial for various cellular processes, including gene regulation, DNA repair, and replication. Additionally, ACTL6A can enhance cancer cell survival even when functioning independently of the SWI/SNF complex.^20^ Secondly, the INO80 chromatin remodeling complex, also ATP-dependent, plays a role in DNA repair, replication, and regulation of gene expression.^21^,^22^ Within this complex, ACTL6A acts as a subunit that facilitates chromatin remodeling, helping to regulate gene expression and maintain genome stability.^23^ Thirdly, the NuA4/TIP60 complex is an acetyltransferase complex responsible for the acetylation of histone H4 and H2A, which impacts gene expression, DNA repair, and cell cycle progression.^24^ ACTL6A is a subunit in this complex that regulates acetyltransferase activity, promoting chromatin accessibility and facilitating gene expression.^25^ Moreover, ACTL6A is vital for maintaining stem cell characteristics and self-renewal capacity by regulating chromatin structure. It is essential to preserve the undifferentiated state of stem cells and progenitor cells.^16^

**Figure 1:**
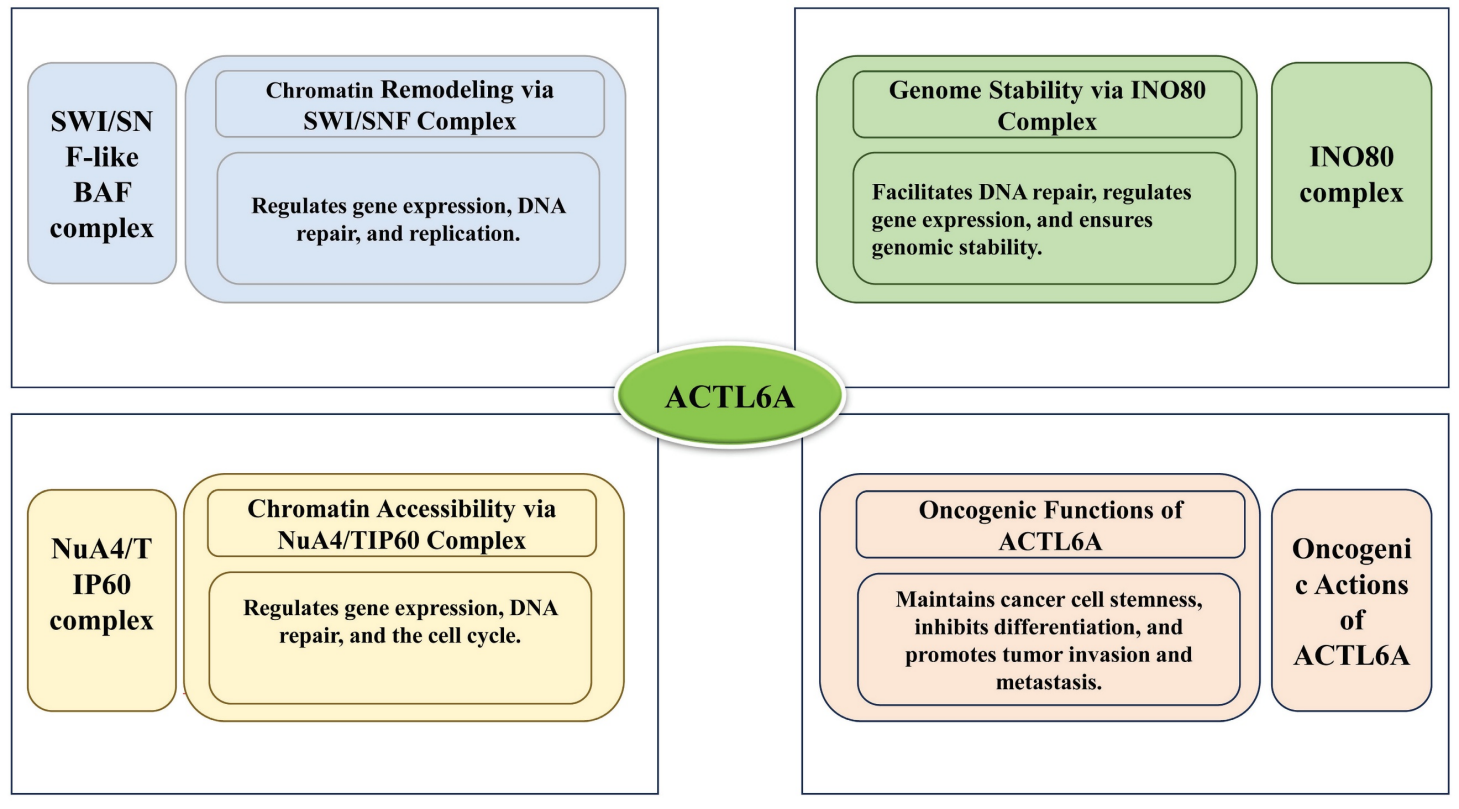
The roles of ACTL6A in different chromatin-associated complexes and its oncogenic functions.

As a central component of chromatin remodeling complexes, ACTL6A is involved in numerous biological processes, including chromatin remodeling, gene expression regulation, stem cell maintenance, cellular differentiation, embryonic development, DNA repair, and cancer. ACTL6A broadly impacts cellular functions and developmental processes by modulating chromatin structure and gene expression. Understanding the specific mechanisms of ACTL6A is crucial for deciphering gene regulatory networks and the molecular basis of various diseases. As shown in Figure 1, ACTL6A functions as a key component of chromatin remodeling complexes, including SWI/SNF, INO80, and NuA4/TIP60, while also exerting independent oncogenic effects by promoting cancer cell stemness, invasion, and metastasis.

## The Role of ACTL6A in Normal Physiological Processes

ACTL6A is a critical component of the SWI/SNF chromatin-remodeling complex. As an essential regulator of cellular and physiological processes, ACTL6A maintains normal cellular functions, such as chromatin remodeling,^23^ cell cycle progression and differentiation,^26^,^27^ muscle regeneration,^28^ and immune system function through various mechanisms.^29^

### Maintaining chromatin structure and promoting gene transcription

ACTL6A plays a pivotal role in maintaining chromatin structure within the nucleus.^30^ It interacts with various associated proteins to regulate the positioning of nucleosomes and accessibility of chromatin, which in turn affects gene activation and transcriptional regulation.^23^ These functions are essential for cell type-specific gene expression in different cell types and for sustaining normal cellular activities.

### Regulation of the cell cycle and differentiation

ACTL6A is vital for ensuring proper cell cycle progression by influencing the expression of related genes, thereby coordinating both cell cycle arrest and progression.^27^ Studies have demonstrated that ACTL6A prevents the SWI/SNF complex from binding to the promoters of KLF4 and other genes associated with differentiation.^31^ The catalytic subunits of the SWI/SNF complex are essential for activating KLF4-regulated target genes, and this activation depends entirely on these catalytic subunits. By sequestering the SWI/SNF complex, ACTL6A inhibits differentiation programs, thus maintaining the progenitor state of epidermal stem cells.^26^ Moreover, ACTL6A is vital for maintaining embryonic stem cells (ESCs) .^16^ It prevents mouse ESCs (mESCs) from differentiating into primitive endoderm (PrE). When ACTL6A is knocked down, mESC begins to differentiate into PrE, while overexpressing ACTL6A suppresses this differentiation. ACTL6A interacts with Nanog and Sox2 to enhance Nanog's binding to pluripotency genes and to reduce the expression of key regulators involved in PrE, effectively preventing mESCs from transitioning into the PrE lineage.

Additionally, ACTL6A is involved in neuronal and muscle differentiation. It has been shown to disrupt differentiation processes in specific muscle disease models.^32^ For example, ACTL6A levels are significantly higher in primary rhabdomyosarcoma (RMS) tumors than in normal muscle tissue, and it is directly targeted by miR-206. Sustained expression of ACTL6A in muscle cells hampers differentiation, while silencing ACTL6A in RMS cells increases the expression of muscle markers, inhibits cell proliferation, and suppresses tumor growth.^33^

### Muscle function and regeneration

In the development and regeneration of skeletal muscle, ACTL6A plays a crucial role in regulating the expression of muscle-specific genes. 33 It is essential for muscle cell differentiation and recovery following injury. The proteins BRG1, BAF47, and ACTL6A are all expressed in proliferating C2C12 myoblasts, with ACTL6A maintaining a stable level during differentiation.^28^ In contrast, the levels of BRG1 and BAF47 decline as the muscle undergoes terminal differentiation. Moreover, dysregulation of ACTL6A has been linked to specific muscle atrophy diseases, highlighting its potential role in muscle pathology. Studies using small molecule inhibitors, such as PFI-3, have demonstrated that inhibiting the bromodomain function of the mSWI/SNF complex in C2C12 myoblasts reduces differentiation efficiency, increases proliferation, and alters the expression of muscle-specific genes.^34^ These findings emphasize the critical role of ACTL6A in the differentiation and regeneration of skeletal muscle.

### Role in the immune system

ACTL6A is essential for developing and functioning immune cells, including T and B cells.^35^ It is critical in the precise transcriptional control necessary for immune cell maturation and infection resistance. During the activation of lymphocytes, antigen receptor signaling quickly facilitates the binding of BAF complexes to chromatin, a process that PIP2 regulates. Importantly, BAF complexes containing β-actin and the actin-related protein BAF53 (ACTL6A) are vital for ATPase activity and interaction with chromatin.^29^ These findings establish a direct link between membrane signaling and chromatin regulation, underscoring the role of the SWI/SNF or BAF complex in mammalian signal transduction. Understanding the biological mechanisms underlying ACTL6A offers valuable insights into human health and the pathogenesis of diseases.

## The Oncogenic Role of ACTL6A across Various Cancers

The study of ACTL6A across cancer types highlights its crucial function as an oncogene in multiple malignancies. This overview includes ACTL6A studies in liver, squamous cell carcinoma, breast, glioblastoma, gastric, colorectal, ovarian, cervical, and other cancers. Table 1 summarizes key studies on ACTL6A across various cancer types, highlighting its role as an oncogene and its involvement in tumor progression, metastasis, and poor prognosis in liver, squamous cell carcinoma, breast, glioblastoma, gastric, colorectal, ovarian, cervical, and other malignancies.

### ACTL6A and liver cancer

Liver cancer is a major cause of cancer-related deaths globally, with HCC being the most common type, representing 70% to 85% of all liver cancer cases.^36^ It is classified into HCC, intrahepatic cholangiocarcinoma (ICC), and mixed hepatocellular cholangiocarcinoma.^37^

### ACTL6A and HCC

HCC typically arises in individuals with chronic liver diseases, such as hepatitis B and C infections or cirrhosis.^38^ Unfortunately, HCC often shows few symptoms in its early stages, which results in many patients being diagnosed only at advanced stages. This late diagnosis limits treatment options and leads to a poor prognosis.^39^ ACTL6A has been identified as a key biomarker driving HCC progression.

Clinical cohort analysis of TCGA data demonstrates that ACTL6A expression levels are significantly elevated in HCC tissues compared to adjacent non-tumor tissues (p<0.001).^40^ High ACTL6A expression correlates significantly with tumor nodule number (p=0.011), vascular invasion (p=0.006), and metastasis development (p=0.01).^41^,^42^ Notably, ACTL6A expression levels hold significant prognostic value: patients with high expression exhibited 1-year, 3-year, and 5-year overall survival (OS) rates of 80.8%, 38.1%, and 21.9% respectively, which were significantly lower than the 96.3%, 73.5%, and 51.0% observed in the low-expression group (P=0.003). Disease-free survival (DFS) also showed marked differences (5-year DFS: 9.1% in high-expression vs. 18.5% in low-expression groups, P=0.019). 42 Multivariate Cox regression analysis confirmed ACTL6A as an independent prognostic factor for HCC (HR=2.775, 95%CI 1.301-5.918, p=0.008).^42^

At the molecular mechanism level, ACTL6A influences HCC progression through a multidimensional regulatory network (Figure 1). Basic research has revealed that it promotes epithelial-mesenchymal transition (EMT) via the SOX2/Notch1 signaling axis, increasing HCC cell invasion capacity.^42^ Notably, Wang *et al.*^10^,^43^,^44^ first identified the regulatory role of E3 ubiquitin ligase FBXW7 on ACTL6A: in HCC tissues, FBXW7 expression negatively correlated with ACTL6A. FBXW7 reduces ACTL6A protein half-life through ubiquitination-mediated degradation, significantly inhibiting cancer stem cell properties. However, the recently reported VPS72-ACTL6A-MYC regulatory axis by Liu *et al.*^14^ suggests a non-ubiquitination-dependent mechanism: this complex enhances MYC transcriptional activity (dual-luciferase reporter assays confirmed that knockdown of VPS72 significantly reduced MYC-specific E-box element activity in HCC cells (p<0.01), whereas VPS72 overexpression increased E-box activity (p<0.01)), promotes glycolysis-related gene expression (HK2, LDHA (p<0.01)), and contributes to HCC progression through metabolic reprogramming.

**Figure 2:**
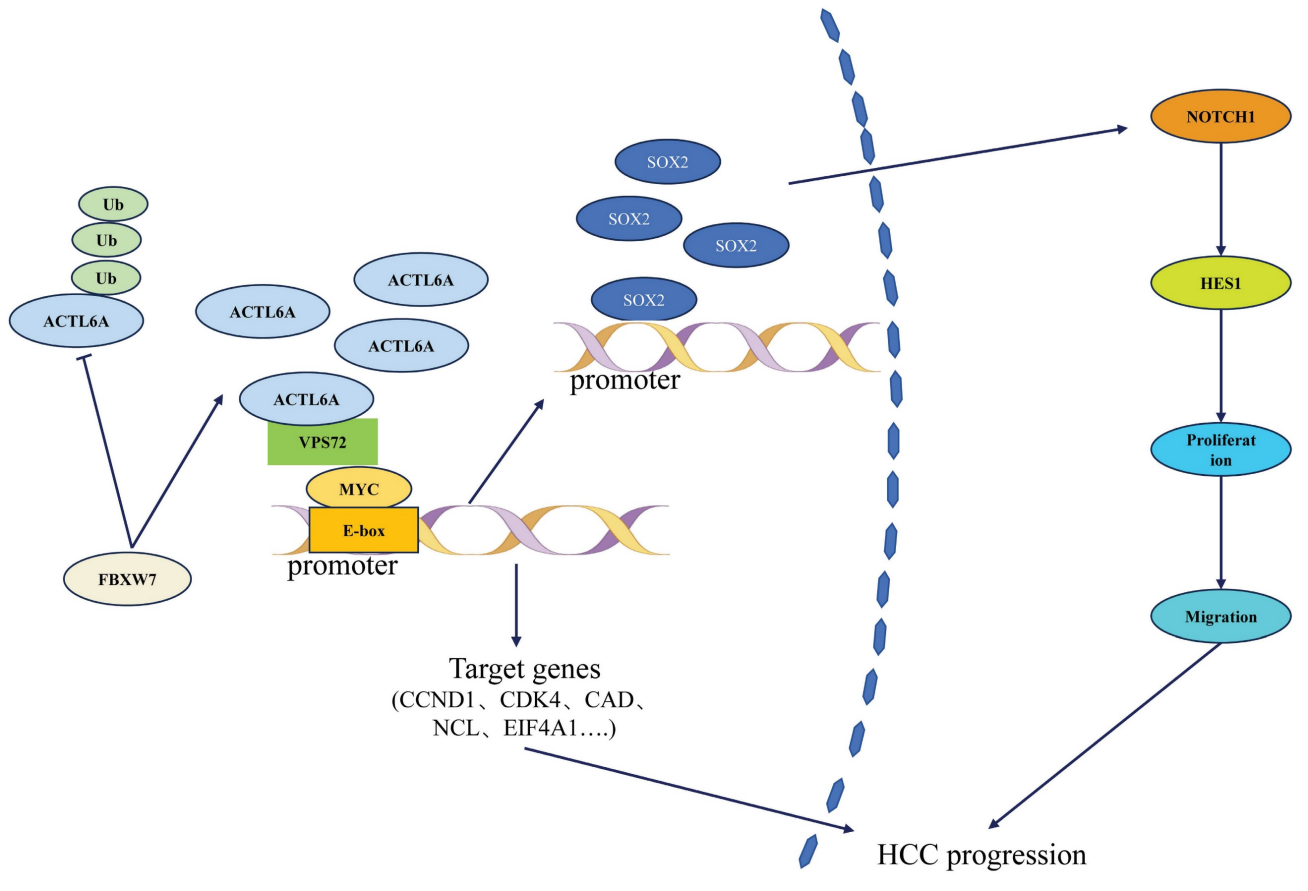
Mechanism of ACTL6A in HCC.

As shown in Figure 2, ACTL6A regulates HCC progression through interactions with key factors such as FBXW7, VPS72, MYC, and the Notch1 signaling pathway.

In conclusion, ACTL6A drives HCC progression through multiple mechanisms including epigenetic reprogramming and metabolic regulation. Although existing studies have revealed its potential as both a prognostic biomarker and therapeutic target, challenges such as tissue-specific regulatory networks and targeted delivery efficiency remain unresolved. Future research should prioritize the development of ACTL6A-based molecular subtyping for individualized treatment strategies and explore its synergistic effects with immune checkpoint inhibitors.

### ACTL6A and cholangiocarcinoma

Cholangiocarcinoma (CCA), a highly aggressive tumor originating from biliary epithelium, is closely associated with chromatin remodeling abnormalities.^45^-^47^ Recent studies have identified that ACTL6A, a subunit of the SWI/SNF chromatin remodeling complex, plays a critical role in CCA through modulating oncogenic signaling pathways.^48^,^49^ Papoutsoglou *et al.*^49^ first uncovered the interaction mechanism between ACTL6A and long noncoding RNA LINC00313: under TGFβ signaling activation, LINC00313 recruits the SWI/SNF complex (containing ACTL6A and BRG1) to the promoter regions of TCF7 and SULF2 genes, significantly enhancing the expression of TCF7, a key transcription factor in the Wnt pathway, thereby activating TCF/LEF-dependent transcription. Functional experiments demonstrated that this regulatory axis promotes colony formation ability of CCA cells and accelerates tumor growth in mouse xenograft models. Notably, ACTL6A not only acts as an effector molecule for chromatin remodeling in this process but also coordinates epigenetic reprogramming by maintaining the structural stability of the SWI/SNF complex. This finding provides direct evidence for understanding the dual roles of ACTL6A (signaling pathway regulation and chromatin remodeling) in CCA malignant progression and suggests that targeting the ACTL6A-LINC00313 interaction may represent a novel strategy to interfere with Wnt/TGFβ crosstalk.

### ACTL6A and squamous cell carcinoma

Squamous cell carcinoma (SCC), a highly aggressive tumor originating from stratified squamous epithelium, is closely associated with epigenetic regulatory abnormalities.^50^-^52^ Recent studies indicate that ACTL6A, a core subunit of the SWI/SNF chromatin remodeling complex, exerts oncogenic effects in SCC through integrating multiple signaling pathways, with its molecular mechanisms demonstrating significant complexity.^48^,^53^

### Collaboration between chromatin remodeling and oncogenic complexes

ACTL6A gene amplification represents a critical early event in SCC development.^48^ Its overexpression promotes BAF complex assembly, enhances its antagonistic capacity against the polycomb complex, and thereby activates oncogene transcription.^48^ Notably, ACTL6A mediates the chromatin co-localization of both BAF and TEAD-YAP complexes, forming a "molecular AND gate" that initiates and maintains SCC malignant characteristics.^48^ This dual regulatory mechanism reveals the pivotal role of ACTL6A in cross-regulating epigenetic and transcription factor networks.

### Bidirectional regulation of p53 signaling pathway

The interaction between ACTL6A and p53 exhibits multimodal characteristics: On one hand, Suruchi Shrestha *et al.*^54^ found that ACTL6A suppresses p21^Cip1 transcription by occupying p53-binding sites (p53-1/p53-2) and Sp1 domains in the p21^Cip1 gene promoter, thereby promoting SCC cell proliferation, invasion, and tumorigenesis—an effect independent of TP53 expression status. On the other hand, ACTL6A enhances p53 acetylation levels, weakening its binding to the p21^Cip1 promoter and indirectly inhibiting growth arrest function.^55^ This "double-edged" regulation of p53 signaling suggests that ACTL6A may reprogram tumor suppressor pathways through epigenetic modifications.

### Organ-specific regulation and clinical translation

In laryngeal squamous cell carcinoma (LSCC), ACTL6A significantly enhances tumor proliferation and invasion by activating the YAP signaling pathway.^53^ *In vitro* and *in vivo* experiments confirmed that silencing ACTL6A inhibits YAP pathway activity and reduces tumor burden, while YAP agonists reverse the phenotypic suppression caused by ACTL6A deficiency.^53^ In oral squamous cell carcinoma (OSCC), high ACTL6A expression correlates with TP53 mutation enrichment, aberrant E2F7/TP63 transcription factors, and immune microenvironment remodeling—its expression level serving as an independent prognostic indicator.^56^ Notably, ACTL6A demonstrates a "dose-effect" relationship in both LSCC and OSCC, where expression levels positively correlate with malignancy degree, suggesting its potential as a shared therapeutic target across organ-specific SCCs.

### ACTL6A and breast cancer

Breast cancer, a common malignancy in women, consists of various subtypes, including hormone receptor-positive, HER2-positive, and triple-negative breast cancer (TNBC), with TNBC having a poor prognosis and high metastasis risk.^57^-^59^

As a core subunit of the SWI/SNF chromatin remodeling complex, ACTL6A exhibits significant pro-tumorigenic properties in breast cancer, particularly in aggressive subtypes like TNBC.^60^,^61^ Clinical cohort analysis demonstrates that high ACTL6A expression correlates with poor patient prognosis.^62^ Its overexpression drives tumor progression through a dual mechanism: first, ACTL6A directly binds to the oncoprotein MYC and inhibits GSK3β-mediated Thr58 phosphorylation, thereby blocking the ubiquitination-dependent degradation pathway of MYC and leading to its aberrant stabilization.^60^ Second, stabilized MYC further recruits histone acetyltransferase KAT5 to the CDK2 promoter region, forming an "ACTL6A-MYC-KAT5" transcriptional complex that triggers excessive CDK2 activation and accelerates G1/S cell cycle transition, ultimately promoting TNBC cell proliferation and *in vivo* tumorigenesis.^60^ Notably, beyond cell cycle regulation, ACTL6A also exacerbates chemoresistance in breast cancer by maintaining cancer stem cell self-renewal and promoting EMT, potentially through epigenetic reprogramming of stemness factors (e.g., SOX2/OCT4) and miR-543-mediated regulatory pathways.^16^,^62^,^63^ Although targeting downstream effectors of ACTL6A (e.g., CDK2 inhibitors combined with paclitaxel) has shown synergistic antitumor effects in mouse models, current research faces critical limitations: first, no small-molecule inhibitors or degraders directly targeting ACTL6A have been developed, hindering clinical translation; second, the functional heterogeneity of ACTL6A in HER2-positive and other subtypes remains unclear, necessitating further exploration of subtype-specific regulatory networks using single-cell sequencing and organoid models.^60^ These challenges highlight the need for future studies to deepen the translational value of ACTL6A across three dimensions: "molecular mechanisms-subtype differences-precision intervention.

### ACTL6A and glioblastoma

Glioblastoma (GBM) is the most common and aggressive malignant brain tumor, marked by high heterogeneity, rapid growth, extensive infiltration, frequent recurrence, and resistance to standard therapies.^64^-^67^ Despite advances in surgery, radiotherapy, and chemotherapy, the median survival for GBM patients remains only 12-15 months.^68^,^69^ Its pathogenesis involves multiple signaling pathways, with cell cycle regulation and anti-apoptotic mechanisms playing key roles in tumor progression.^70^

ACTL6A drives malignant progression of GBM through epigenetic remodeling (cell cycle and apoptosis-related genes) and crosstalk with critical signaling pathways.^71^ Clinical evidence shows that ACTL6A expression is significantly higher in GBM tissues compared to normal brain tissues, with its high expression closely associated with shortened median survival time and enhanced cancer stem cell properties in patients.^72^ At the molecular mechanism level, ACTL6A forms a complex with Hippo pathway effectors YAP/TAZ, specifically blocking β-TrCP E3 ubiquitin ligase recognition of YAP and thereby inhibiting YAP ubiquitination-dependent degradation.^15^ This aberrantly stabilized YAP further activates downstream pro-tumorigenic transcriptional programs, leading to overexpression of cell cycle regulatory genes (e.g., Cyclin D1) and anti-apoptotic proteins (e.g., Bcl-2), ultimately promoting GBM cell proliferation and treatment resistance.^71^ Notably, ACTL6A also enhances AKT signaling activation through a Hippo-independent pathway: experiments confirm that ACTL6A knockdown significantly reduces phosphorylated AKT (Ser473) levels, thereby inhibiting GBM cell migration and invasion capabilities.^72^ Clinically more importantly, targeted silencing of ACTL6A significantly improves GBM cell sensitivity to temozolomide (TMZ), with its sensitizing effect likely related to reprogramming of DNA damage repair pathways (e.g., MGMT expression regulation).^72^ Collectively, these findings establish ACTL6A as a core regulatory node of "stemness-drug resistance" in GBM, providing a novel direction for developing epigenetic-chemotherapy combination strategies.

### ACTL6A and gastric cancer

Gastric cancer (GC) is a common and deadly malignancy, remaining a leading cause of cancer-related mortality.^73^,^74^ Despite recent advances in diagnosis and treatment, its aggressive nature, high metastatic potential, and lack of early symptoms often lead to late-stage diagnoses and poor prognosis.^75^ The development of GC involves multiple signaling pathways and molecular mechanisms.^76^ Recently, researchers have focused on identifying molecular markers associated with GC invasion and metastasis, with ACTL6A receiving significant attention.

High expression of ACTL6A is closely associated with invasion and metastasis capabilities in GC, with its pro-tumorigenic mechanisms involving a unique metabolic regulatory network. Using stable isotope labeling combined with chromatin immunoprecipitation (ChIP) technology, Yang *et al.*^77^ revealed that ACTL6A acts as a co-transcription factor directly binding to the promoter region of glutamate-cysteine ligase catalytic subunit (GCLC), significantly upregulating this rate-limiting enzyme for glutathione (GSH) synthesis. The resulting GSH metabolic reprogramming synergistically maintains the malignant phenotype of GC through dual effects: on one hand, GSH inhibits ferroptosis by neutralizing lipid peroxides, protecting GC cell survival in metastatic microenvironments; on the other hand, GSH metabolic byproducts (e.g., cysteine/glutamate) activate matrix metalloproteinases such as MMP-9, promoting extracellular matrix degradation and distant metastasis. Animal experiments further confirmed that ACTL6A silencing significantly reduced lung metastasis burden of orthotopic xenografts, accompanied by decreased GSH/GSSG ratios and accumulated lipid peroxidation markers (e.g., MDA) in tumor tissues.^77^ Notably, targeted intervention of the GSH synthesis pathway combined with standard chemotherapy drugs (e.g., BSO) synergistically induced GC cell death, providing a novel strategy to overcome chemoresistance in gastric cancer.

### ACTL6A and colorectal cancer

Colorectal cancer (CRC) is a leading global malignancy with rising incidence and mortality rates.^78^,^79^ Its development is driven by the dysregulation of key molecular pathways, including Wnt/β-catenin,^80^ PI3K/Akt,^81^ and MAPK.^82^ Recent studies have identified abnormal expression of specific molecular markers in CRC that are closely linked to these aggressive behaviors.^83^

High ACTL6A expression directly correlates with advanced CRC progression, including deeper tumor invasion (pT), distant metastasis, poor differentiation, and microvascular/perineural invasion.^84^ ACTL6A exhibits multi-dimensional oncogenic regulation in CRC. Beyond its well-documented roles in promoting tumor cell proliferation, migration, and EMT, mechanistic studies by Yang *et al.*^85^ uncovered its core function in activating the MAPK signaling pathway through epigenetic remodeling. Specifically, ACTL6A forms a complex with transcriptional repressor P63, directly binding to the promoter region of phosphatase DUSP5 to suppress its transcription, resulting in loss of DUSP5 protein expression. As a negative regulator of ERK1/2 phosphorylation, DUSP5 deficiency leads to sustained phosphorylation of ERK1/2, thereby driving aberrant activation of the MAPK signaling cascade. This regulatory cascade manifests clinically as a significant association between high ACTL6A expression and increased CRC liver metastasis rates, shortened progression-free survival in patients. Notably, MAPK activation not only accelerates cell cycle progression but also reinforces EMT phenotypes through upregulation of transcription factors such as SNAIL, forming a "proliferation-invasion" positive feedback loop. Based on these findings, targeted silencing of ACTL6A could synergistically inhibit both MAPK signaling and EMT progression, providing a novel therapeutic direction by combining blockade of metastatic microenvironment and intracellular signaling pathways.

### ACTL6A and ovarian cancer

Ovarian cancer is a malignancy of the female reproductive system, often diagnosed at advanced stages due to the lack of early symptoms.^86^-^89^

One critical metabolic feature of ovarian cancer is its reliance on the glycolytic pathway for energy production, particularly in rapidly dividing tumor cells. 90-93

ACTL6A has been identified as a key regulator of glycolysis in ovarian cancer. 11,94 By upregulating phosphoglycerate kinase 1 (PGK1), ACTL6A enhances glycolysis triggered by follicle-stimulating hormone (FSH), promoting tumor growth and metastasis. The findings clarify the role of ACTL6A in metabolic reprogramming in ovarian cancer and propose a potential molecular mechanism for targeting ACTL6A to inhibit glycolysis and exert anti-tumor activity.

Building upon its prometastatic roles in CRC, ACTL6A also exhibits significant pro-invasive and chemoresistant properties in ovarian cancer. Multi-omics integrated analysis identified ACTL6A as a core member of invasion-associated gene signatures, with its high expression strongly correlated with shortened overall survival in ovarian cancer patients.^95^ Gene set enrichment analysis (GSEA) further revealed that ACTL6A coordinately promotes tumor cell proliferation and matrix detachment through dual regulation: positive modulation of the "cell cycle" pathway and negative regulation of the "focal adhesion" pathway. Notably, drug sensitivity prediction modeling uncovered selective inhibitory effects of HER2 inhibitors (e.g., CP724714) against ACTL6A-high ovarian cancer cells, suggesting that targeting receptor tyrosine kinase signaling may overcome ACTL6A-mediated chemoresistance.

Collectively, these findings establish ACTL6A as a dual-functional target for both prognostic assessment and precision therapy in ovarian cancer.

### ACTL6A and cervical cancer

Cervical cancer progression involves complex molecular interactions, with ACTL6A emerging as a critical oncoprotein in this malignancy.^96^-^98^ Functional studies revealed that ACTL6A promotes cervical cancer progression through oncogenic c-Myc interaction, where it stabilizes c-Myc protein levels and enhances its transcriptional activity. This interaction upregulates cell cycle regulators such as Cyclin D1 and CDK2, driving aberrant proliferation and tumorigenesis.^96^ Clinically, ACTL6A overexpression correlates with advanced disease stages and poor patient outcomes, underscoring its prognostic significance.

However, recent studies have uncovered a counter-regulatory mechanism involving microRNA-216a-3p (miR-216a-3p), which acts as a tumor suppressor by targeting ACTL6A.^99^ miR-216a-3p levels inversely correlate with ACTL6A expression in cervical cancer tissues, and its overexpression inhibits cell proliferation, invasion, and EMT.^99^ Mechanistically, miR-216a-3p directly binds to the ACTL6A 3'-UTR, reducing its protein abundance. This reduction disrupts the ACTL6A-YAP interaction, leading to YAP phosphorylation and inactivation of its downstream oncogenic programs (e.g., CTGF and Cyr61 expression). Rescue experiments confirmed that restoring ACTL6A expression or reactivating YAP signaling abrogates miR-216a-3p-mediated tumor suppression, establishing a functional axis: miR-216a-3p→ACTL6A→YAP.

Collectively, these findings demonstrate a dual role for ACTL6A in cervical cancer: as a prometastatic effector downstream of miR-216a-3p loss and as a critical node linking epigenetic regulation to YAP oncogenic signaling. Targeting this regulatory circuit may provide innovative therapeutic opportunities for cervical cancer patients with ACTL6A-driven disease.

### ACTL6A and other cancers

RMS is an aggressive pediatric soft tissue sarcoma characterized by rapid growth and poor differentiation.^100^,^101^ Studies have shown that ACTL6A is significantly overexpressed in RMS tumors compared to normal muscle tissue, highlighting its potential role in RMS development.^33^ ACTL6A has been identified as a direct target of miR-206, and its persistent expression in myoblasts disrupts normal muscle differentiation.^102^ Knocking out ACTL6A in RMS cells leads to increased expression of muscle markers, decreased cell proliferation, and reduced anchorage-independent growth. Moreover, ACTL6A knockout suppresses tumor growth in both embryonal and alveolar RMS models, promoting the morphological and biochemical differentiation of these tumors. These findings suggest that abnormal expression of ACTL6A is crucial in the pathogenesis of RMS and may serve as a potential therapeutic target for the disease.

In the context of lung cancer, particularly non-small cell lung cancer (NSCLC),^103^,^104^ ACTL6A has been associated with poor prognosis. NSCLC is the leading cause of cancer-related deaths worldwide, and its progression is often driven by dysregulated molecular mechanisms, many of which represent potential therapeutic targets.^105^-^108^ High expression of ACTL6A in NSCLC tissues and cells has been linked to worse clinical outcomes, with suppression of ACTL6A expression leading to significant inhibition of NSCLC cell growth and increased apoptosis.^109^,^110^ Mechanistically, ACTL6A overexpression downregulates WWC1, a known inhibitor of YAP activation, in NCI-H2170 cells, while ACTL6A-targeting shRNA increases WWC1 expression. Furthermore, ACTL6A overexpression also leads to the upregulation of YAP, TAZ, and CYR61 proteins, suggesting that ACTL6A activates the Hippo/YAP signaling pathway. These findings underscore the pivotal role of ACTL6A in the progression of NSCLC and indicate that it could be a promising therapeutic target for this malignancy.

Together, these studies highlight the potential of ACTL6A as a key regulator in the development and progression of RMS and NSCLC, making it an attractive target for therapeutic intervention in these cancers.

### Clinical applications

Accumulating evidence has shown that the overexpression of ACTL6A plays a critical role in resistance to platinum-based chemotherapy^62^,^111^. Specifically, increased levels of ACTL6A enhance the repair of cisplatin-DNA adducts after cisplatin treatment, contributing to cisplatin resistance. In contrast, depleting ACTL6A hampers the repair of DNA damage caused by cisplatin, thereby making platinum-resistant ovarian cancer cells more sensitive to the drug. The SWI/SNF chromatin remodeling complex is responsible for regulating this repair process. Additionally, studies using xenograft mouse models have shown that treatment with histone deacetylase (HDAC) inhibitors can counteract the effects of ACTL6A overexpression on the repair of cisplatin-induced DNA damage repair, ultimately sensitizing cancer cells to cisplatin.^111^ These findings highlight a novel role for ACTL6A in platinum resistance and suggest a potential strategy for utilizing HDAC inhibitors in treating platinum-resistant tumors.

ACTL6A plays a crucial role in several types of cancer, making it a promising target for therapeutic strategies. For example, developing small molecule inhibitors or siRNA-based technologies aimed at ACTL6A could effectively reduce tumor cell proliferation and metastasis. Crucially, the natural compound sulforaphane demonstrates therapeutic potential by targeting this pathway to suppress glioma cell growth, positioning ACTL6A as both a prognostic biomarker and a druggable target for glioblastoma treatment.^112^ Furthermore, targeting the signaling pathways associated with ACTL6A in conjunction with existing chemotherapy or targeted therapies may enhance treatment effectiveness and help overcome resistance.^12^

Although promising preclinical results exist, several challenges still exist for therapies targeting ACTL6A. These challenges include ensuring that the treatments are specific and safe to prevent off-target effects in normal cells, overcoming cancer resistance, and collecting robust clinical evidence to support their use in patients.

Future research should clarify the specific mechanisms of ACTL6A in various tumor types and investigate its interactions with other molecular markers. This helps to define therapeutic targets and develop more effective treatment strategies. Utilizing advanced biotechnological and drug design approaches is crucial for creating targeted and safe drugs focusing on ACTL6A, which could provide new therapeutic opportunities for cancer patients.

## Conclusion

As a key chromatin remodeling factor, ACTL6A plays a crucial role in the development and progression of various cancers. Detailed studies of ACTL6A's mechanisms across different tumor types may lead to new diagnostic and therapeutic strategies. Further studies are needed to fully understand its functions and mechanisms in cancer, which will help establish a foundation for its clinical applications.

## Figures and Tables

**Table 1:** Summary of studies on ACTL6A in various cancers

Article Title	Cancer Type	Expression Level & Functional Role	Molecular Mechanism	Journal (Year)	Reference	
FBXW7 Reduces the Cancer Stem Cell-Like Properties of Hepatocellular Carcinoma by Regulating the Ubiquitination and Degradation of ACTL6A.	Hepatocellular Carcinoma	Upregulation, oncogene	FBXW7 inhibits the activity of ACTL6A, thereby suppressing HCC cell growth, proliferation, and drug resistance.	Stem Cells Int.(2022)	^10^	
ACTL6A regulates follicle-stimulating hormone-driven glycolysis in ovarian cancer cells via PGK1.	Ovarian Cancer	Upregulation, oncogene	Regulates glycolysis by affecting PGK1 expression and promots cell proliferation and migration.	Cell Death Dis.(2019)	^11^	

ACTL6A interacts with p53 in acute promyelocytic leukemia cell lines to affect differentiation via the Sox2/Notch1 signaling pathway.	Acute Promyelocytic Leukemia	Upregulation, oncogene	Interacts with Sox2 and P53, suppressing differentiation through the Sox2 and Notch1 signaling pathways.	Cell.Signal.(2019)	^13^	

Targeting VPS72 inhibits ACTL6A/MYC axis activity in HCC progression.	Hepatocellular Carcinoma	Upregulation, oncogene	VPS72 interacts with MYC and ACTL6A, facilitating the formation of the ACTL6A/MYC complex. The interaction between VPS72 and ACTL6A enhances MYC's affinity for its target gene promoters and promotes downstream target gene transcription, advancing HCC progression.	Hepatology(2023)	^14^	
Actin like-6A promotes glioma progression through stabilization of transcriptional regulators YAP/TAZ.	Glioma	Upregulation, oncogene	Binds to YAP/TAZ proteins, preventing YAP protein degradation, thereby activating downstream signaling pathways and promoting tumor cell proliferation, migration, and invasion.	Cell Death Dis.(2018)	^15^	
ACTL6A Is Co-Amplified with p63 in Squamous Cell Carcinoma to Drive YAP Activation, Regenerative Proliferation, and Poor Prognosis.	Head and Neck Squamous Cell Carcinoma	Upregulation, oncogene	ACTL6A and P63 inhibit WWC1, thereby activating YAP, promoting tumor growth, and suppressing differentiation.	Cancer Cell(2017)	^20^	

Actin-like 6A predicts poor prognosis of hepatocellular carcinoma and promotes metastasis and epithelial-mesenchymal transition.	Hepatocellular Carcinoma	Upregulation, oncogene	Promotes metastasis and EMT through SOX2/Notch1 signaling.	Hepatology(2016)	^42^	

Increased ACTL6A occupancy within mSWI/SNF chromatin remodelers drives human squamous cell carcinoma.	Human Squamous Cell Carcinoma	Upregulation, oncogene	It enhances the interaction between the BAF complex and the TEAD-YAP axis, establishing a feedback loop that maintains chromatin openness and promotes transcription. This process increases ACTL6A levels within the BAF complex, leading to the redistribution of polycomb proteins and the activation of gene expression associated with SCC tumorigenesis.	Mol.Cell.(2021)	^48^	

ACTL6A suppresses p21Cip1 expression to enhance the epidermal squamous cell carcinoma phenotype.	Epidermal Squamous Cell Carcinoma	Upregulation, oncogene	Interacts with the p53 DNA response element in the p21Cip1 promoter, inhibiting p21Cip1 promoter activity as well as its mRNA and protein levels.	Oncogene(2020)	^54^	

Actin-like protein 6A/MYC/CDK2 axis confers high proliferative activity in triple-negative breast cancer.	Triple-Negative Breast Cancer	Upregulation, oncogene	Stabilizes MYC by inhibiting its ubiquitination, promoting cell proliferation and tumor growth.	J.Exp.Clin.Cancer Res.(2021)	^60^	

ACTL6A protects gastric cancer cells against ferroptosis through induction of glutathione synthesis.	Gastric Cancer	Upregulation, oncogene	Functions as a co-transcription factor with NRF2 to upregulate GCLC expression, thereby reducing reactive oxygen species (ROS) levels and inhibiting ferroptosis.	Nat.Commun.(2023)	^77^	

ACTL6A expression promotes invasion, metastasis and epithelial mesenchymal transition of colon cancer.	Colorectal Cancer	Upregulation, oncogene	Promotes EMT in colorectal cancer cells *in vitro*, thereby enhancing migration and invasion.	BMC Cancer(2018)	^84^	

BAF53A drives colorectal cancer development by regulating DUSP5-mediated ERK phosphorylation.	Colorectal Cancer	Upregulation, oncogene	P63 acts as a potential transcriptional repressor of DUSP5. BAF53A interacts with P63, reducing DUSP5 expression and promoting ERK1/2 phosphorylation.	Cell Death Dis.(2022)	^85^	

Potential Role of SWI/SNF Complex Subunit Actin-Like Protein 6A in Cervical Cancer.	Cervical Cancer	Upregulation, oncogene	ACTL6A interacts with c-Myc, activating the c-Myc target gene E2F1and promots cell cycle progression.	Front.Oncol.(2021)	^96^	

ACTL6A promotes repair of cisplatin-induced DNA damage, a new mechanism of platinum resistance in cancer.	Ovarian Cancer & Lung Squamous Cell Carcinoma	Upregulation, oncogene	Overexpression of ACTL6A enhances the repair of cisplatin-DNA adducts, leading to increased resistance to cisplatin treatment.	Proc.Natl.Acad.Sci.U.S.A.(2021)	^111^	
